# Follow-up Care among Medicaid Patients with Schizophrenia treated with Antipsychotics in the Inpatient Setting

**DOI:** 10.36469/9882

**Published:** 2014-10-02

**Authors:** Amanda M. Farr, David M. Smith, Jacqueline A. Pesa, Zhun Cao

**Affiliations:** 1 Truven Health Analytics, Cambridge, MA, USA; 2 Janssen Scientific Affairs, Titusville, NJ, USA

**Keywords:** administrative claims, continuity of care, antipyschotics, medicaid, schizophrenia

## Abstract

**Background:** For patients with schizophrenia, the transition from inpatient hospitalization to outpatient care presents a challenge to providing continuous care. Lapses in care during this time period can result in poor clinical outcomes. To date, there is little information regarding the association between inpatient antipsychotic treatment and outpatient care.

**Objectives:** The objectives of this study were to describe trends in and identify factors associated with post-discharge follow-up outpatient care among Medicaid enrollees with schizophrenia treated with antipsychotics.

**Methods:** Adults administered oral or long-acting injectable antipsychotic medication during a hospitalization for schizophrenia were identified in the linked MarketScan® Hospital Drug and Multi-State Medicaid Databases. Psychiatric-related follow-up outpatient visits within 30 days of discharge were identified from Medicaid claims based on Healthcare Effectiveness Data and Information Set specifications. Kaplan-Meier curves and Cox proportional hazards models were used to describe and analyze time to follow-up visit and to identify patient and hospitalization characteristics associated with follow-up visit.

**Results:** The study sample (N=1,312) had a mean age of 40.5 years and was 57% male. A follow-up outpatient visit was identified among 47% of patients. The proportion of patients with a follow-up visit ranged from 25% in 2005 to 48% in 2010/2011. The Cox proportional hazard model suggests that capitated health plan, attending physician specialty of psychiatry/psychology, and later year of index hospitalization significantly increase the probability of a follow-up visit, while substance-related disorders significantly decrease the probability. Type of antipsychotic received during index hospitalization was not significantly associated with probability of a follow-up visit.

**Conclusion:** While follow-up visit rates have increased over time, this study highlights the ongoing need for improvements in effective linkage to outpatient care for patients hospitalized and treated for schizophrenia, particularly among patients with comorbid substance abuse disorder.

## BACKGROUND

The effective management of many chronic conditions, including schizophrenia, requires continuous care, incorporating both inpatient and outpatient care settings.[1] The transition from inpatient hospitalization to outpatient care has been highlighted as a problematic time during which care often lapses for schizophrenic patients, resulting in poor outcomes.[2-4] Estimates from 2003-2005 suggest that approximately 50% of all discharged Medicaid psychiatric patients do not transition to outpatient care within 30 days of discharge.[5,6] Factors associated with timely follow-up care include receipt of outpatient psychiatric services prior to hospitalization, absence of a substance abuse disorder, and longer inpatient admission.[5,6] Additionally, a strong indicator of follow-up outpatient care is treatment with antipsychotic medication prior to hospitalization, particularly injectable medications.[5] To our knowledge, the relationship between receipt of different types of antipsychotic therapy during an inpatient stay and outpatient follow-up care has not been examined.

This retrospective analysis sought to evaluate the association between type of inpatient antipsychotic treatment and follow-up outpatient care within 30 days post-discharge among Medicaid enrollees with schizophrenia who were hospitalized and received inpatient antipsychotic treatment. The longitudinal nature of the data used in this analysis allowed for the evaluation of trends in rates of follow-up care over an approximate 6-year time period. Additionally, this study sought to confirm factors previously associated with follow-up outpatient care.

## METHODS

### Data Sources

This analysis used data from the Truven Health MarketScan® Hospital Drug Database (HDD) and the MarketScan Multi-State Medicaid Database, which can be linked using the MarketScan Inpatient Drug Link File (IPL). The HDD contains data derived from hospital billing systems and includes detailed information on inpatient diagnoses, procedures, and drug administration from over 500 US hospitals. The Medicaid Multi-State Database contains fully adjudicated and paid Medicaid claims for inpatient and outpatient services, and outpatient prescription drugs for enrollees from 9 to 12 geographically diverse states, depending on the data year. Several hundred hospitals that contribute data to the HDD can be linked to claims in the Medicaid Multi-State Database using the IPL, allowing utilization and outcomes to be tracked following hospitalization discharge. Because this study used only de-identified patient records and did not involve the collection, use, or transmittal of individually identifiable data, Institutional Review Board approval to conduct this study was not necessary.

### Patient Selection

Patients with hospitalization(s) for schizophrenia between July 1, 2005 and May 15, 2011, who were administered an antipsychotic medication during the inpatient stay were identified in the MarketScan IPL. Schizophrenia hospitalizations were defined using International Classification of Diseases, 9$^{th}$ Edition, Clinical Modification (ICD-9-CM) codes 295.1x-295.6x and 295.8x-295.9x in any diagnosis position. Primary diagnosis was not required as the distinction between primary and non-primary diagnosis may be driven by reimbursement rather than clinical factors. Patients administered only short-acting injectable (SAI) antipsychotics during hospitalization were not included in the study sample; administration of typical oral, typical long-acting injectable (LAI), atypical oral, or atypical LAI antipsychotics during an inpatient stay, based on information from the HDD, was required for study inclusion. To ensure an adequate sample of each antipsychotic medication type given the release date of more atypical LAIs in the United States, the following hierarchy was used to choose an index admission for patients with multiple eligible hospitalizations over the study period: admission with atypical LAI, admission with typical LAI, admission with any oral antipsychotic. If there were multiple admissions with administration of the same antipsychotic medication class, one admission was randomly chosen. Patients were required to be at least 18 years old on the index admission date and continuously enrolled in Medicaid 6 months before the index admission date (preperiod) through at least 1 day after the index discharge date (follow-up period). Patients dually-eligible for Medicare, hospitalized for more than 30 days, discharged to a different healthcare facility (i.e., not discharged to the community), or who left against medical advice were excluded.

### Definition of Outcome

The outcome was time from index hospitalization discharge to outpatient follow-up care, defined as the first psychiatric-related outpatient office or community mental health organization visit in the Medicaid claims. A psychiatric-related follow-up outpatient office visit was an office visit with both a psychiatric primary diagnosis (ICD-9-CM 290.xx-314.xx) and mental health-related healthcare service codes (i.e., psychotherapy, behavioral health screenings, meeting with clinical social worker) within 30 days of discharge based on the National Committee for Quality Assurance's (NCQA) Healthcare Effectiveness Data and Information Set (HEDIS) measure specification.[7] HEDIS measures are reportedly used by more than 90% of America's health plans to measure performance on important dimensions of care and service.[8] Community mental health organization visits were identified through bill type or standard place of service fields. Patients were followed up to 30 days after the index discharge date to assess outpatient follow-up care.

### Definition of Independent Variables and Covariates

The primary independent variables in this analysis were type of inpatient antipsychotic treatment received during the index hospitalization (typical oral, atypical oral, typical LAI, atypical LAI) and year of index hospitalization. Types of inpatient antipsychotic administered were defined from drug codes and route of administration information in the HDD. Year of index hospitalization was based on index hospitalization discharge date. Years 2010 and 2011 were combined due to small sample size.

Covariates included patient demographic, baseline clinical and treatment characteristics, and hospital characteristics which were hypothesized or have been shown to be associated with follow-up in previous analyses.[5] Age, sex, race, urbanicity, insurance plan capitation status (fee-for-service vs. capitated), and Medicaid basis of eligibility were measured on the index admission date using enrollment and claims information from the Medicaid database. To capture the state of a patient's health, psychiatric comorbid conditions, Deyo Charlson Comorbidity Index, and number of unique three-digit ICD-9-CM codes were measured from Medicaid claims during the 6 months prior to the index admission date. Patients with an ICD-9-CM diagnosis on one inpatient facility or two inpatient professional or outpatient claims on different days were considered to have the particular comorbid condition. Also measured in the 6-month period prior to the index admission date with Medicaid claims were the types of antipsychotic medications prescribed and filled based on NDC and HCPCS codes; and inpatient, outpatient, emergency department and psychiatric-related utilization, which represent points of contact with the healthcare system. Characteristics of the index hospitalization, including admission source, specialty of the attending physician, which may be associated with aspects of care received, and length of stay were measured using the HDD. Characteristics of the admitting hospital, including region, urbanicity, hospital size based on number of beds, and teaching status were also measured during the index admission. Hospitalization and hospital characteristics may be associated with type of antipsychotic administered and follow-up, as discharge planning may vary due to hospital characteristics.

### Analysis

A Kaplan-Meier curve showing the probability of having a follow-up visit within 30 days post-discharge was produced. Right censoring prior to the end of the 30-day period occurred if Medicaid enrollment ended, the end of available data in the databases was reached, or the patient was readmitted. Additionally, a figure showing the trend in proportion of patients with a follow-up visit or readmission was produced. Patients experiencing both events were categorized based on the event occurring first chronologically. Descriptive summary statistics of patient and hospital characteristics were produced and t-tests and chi-squared tests were used to compare patients with and without a follow-up visit. Cox proportional hazards regression models were fit and used to determine covariates associated with probability of follow-up outpatient visit. Bivariate analyses informed the choice of variables initially included in the multivariable models. A forward selection method was used to identify the significant covariates with forced inclusion of the four variables representing type of antipsychotic treatment administered during hospitalization as the main predictors of interest. Covariates remained in the final model if the p-values for their hazard ratios were <0.05, which was considered statistically significant. No adjustment was made for multiplicity. The adjusted hazard ratios (AHRs) and 95% confidence intervals (CIs) from the final model are reported. Analyses were conducted using SAS 9.3.

## RESULTS

### Study Sample

There were 5,027 schizophrenia-related hospitalizations in the HDD during which an antipsychotic medication was administered. 114 hospitalizations were excluded based on the age criterion and an additional 1,991 based on the continuous enrollment criterion. Applying the remaining inclusion and exclusion criteria, there were 2,469 eligible hospitalizations. After removing hospitalizations of persons insured through commercial plans or Medicare and applying the hierarchy to select a hospitalization for inclusion, the final study sample was comprised of 1,312 unique patients insured through Medicaid. Of the final sample, 1,025 patients had only one eligible hospitalization and therefore the hierarchy of choosing an admission based on index drug type was not applied, and 1,022 had a psychiatric diagnosis as the primary diagnosis on the index hospitalization claim, including 938 patients with a primary diagnosis of schizophrenia.

### Descriptive Results

Less than half (47%) of the 1,312 patients had a psychiatric-related outpatient office or community mental health organization visit within 30 days of discharge. 42% of patients were followed for a full 30 days and had no follow-up visit while 11% were censored during the 30-day period (10% readmission; 1% end of continuous enrollment). The Kaplan-Meier curve for all patients displays the probability of no follow-up visit against the time after discharge (Figure 1). The proportion of patients with follow-up visit ranged from 25% of individuals with an index hospitalization in 2005 to 57% in 2007 (Figure 2). The proportion of patients who were readmitted within 30 days was between 8% and 14% across years. The bivariate analyses suggest that an outpatient follow-up visit occurred less frequently among Hispanic patients; patients enrolled in non-capitated plans; patients with a diagnosis of alcohol or substance abuserelated disorder; patients cared for by physicians of specialties other than psychiatry/psychology; and patients with no antipsychotic treatment in the pre-period. There were no statistically significant differences in antipsychotic treatment during index admission between patients with or without a follow-up visit (Tables 1 and 2). Regarding hospital characteristics, a greater proportion of patients with a follow-up visit were hospitalized in the North Central region and in a hospital with between 200 and 299 beds (Table 3).

**Figure 1.** Kaplan-Meier Curve for Time to Follow-up Visit

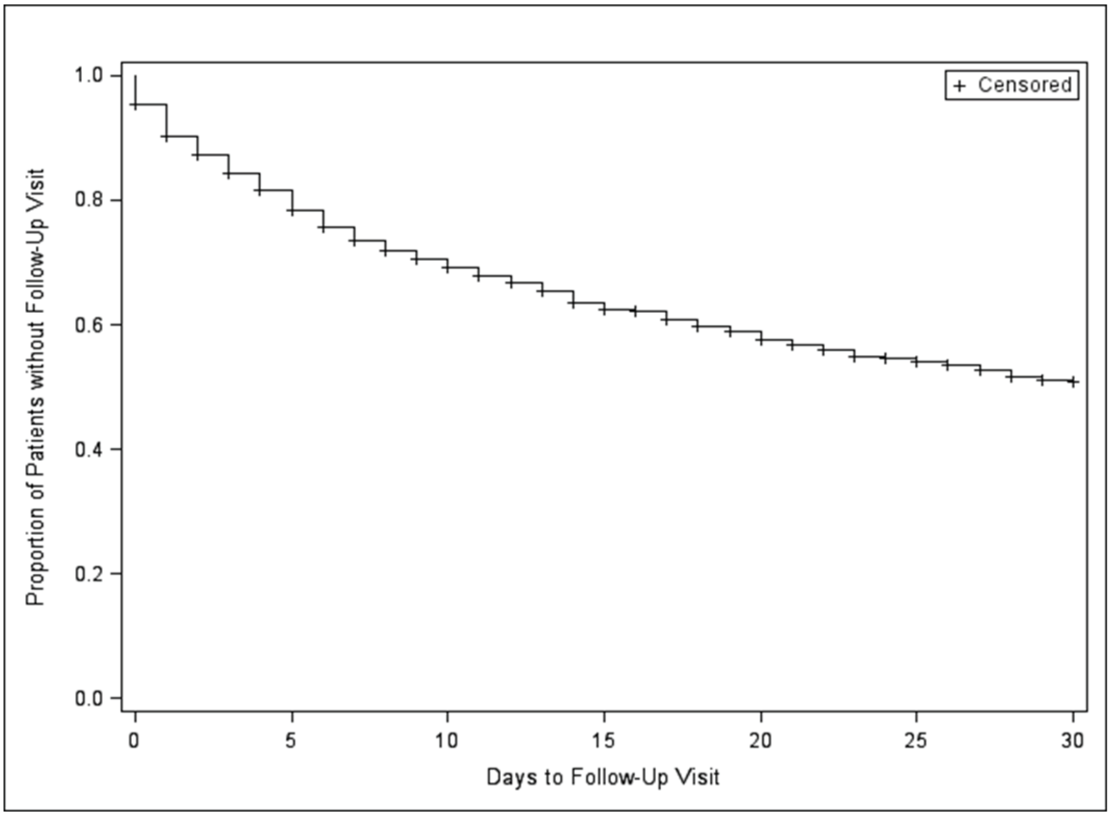

**Figure 2.** Proportion of Patients with an Outpatient Follow-up Visit or Readmission within 30 Days of Discharge by Year

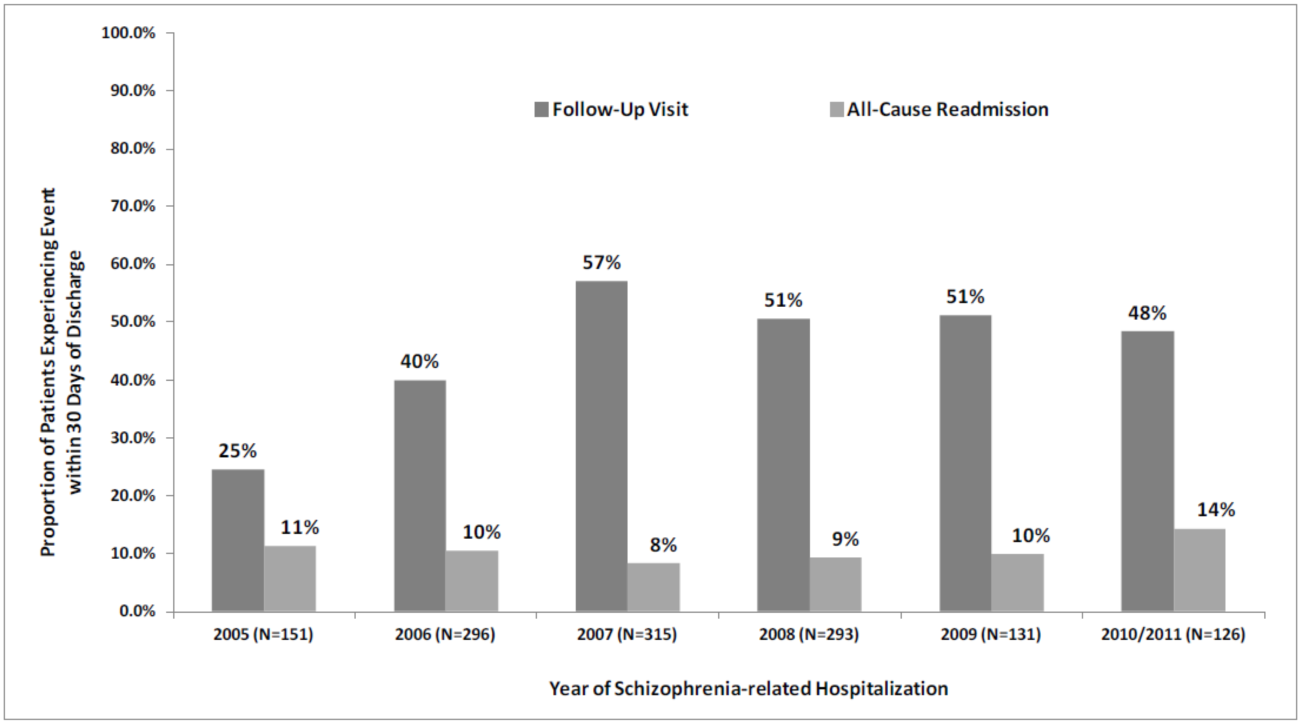

*p-value <0.001; Note: If patients experienced both events, they were categorized based on the event which occurred first chronologically.

**Table 1.** Demographic and Clinical Characteristics

| | All Patients N=1,312 | | Follow-Up Visit N=611 | | No Follow-up Visit N=701 | | |
|---|---|---|---|---|---|---|---|
| | **N/ Mean** | **%/SD** | **N/Mean** | **%/SD** | **N/Mean** | **%/SD** | **P-value** |
| **Age (Mean, SD)** | 40.5 | 13.2 | 39.8 | 13.1 | 41.1 | 13.2 | 0.082 |
| **Sex (N, %)** | | | | | | | 0.982 |
| Male | 691 | 53% | 322 | 53% | 369 | 53% | |
| Female | 621 | 47% | 289 | 47% | 332 | 47% | |
| **Race (N, %)** | | | | | | | <0.001 |
| White | 495 | 38% | 270 | 44% | 225 | 32% | |
| Black | 674 | 51% | 284 | 47% | 390 | 56% | |
| Hispanic | 11 | 1% | 2 | 0% | 9 | 1% | |
| Other/Unknown | 132 | 10% | 55 | 9% | 77 | 11% | |
| **Urbanicity (N, %)** | | | | | | | 0.795 |
| Urban | 1167 | 89% | 542 | 89% | 625 | 89% | |
| Rural | 145 | 11% | 69 | 11% | 76 | 11% | |
| **Insurance Plan (N, %)** | | | | | | | 0.035 |
| Capitated | 64 | 5% | 38 | 6% | 26 | 4% | |
| Non-capitated | 1248 | 95% | 573 | 94% | 675 | 96% | |
| **Paranoid Schizophrenia (N, %)** | 507 | 39% | 254 | 42% | 253 | 36% | 0.042 |
| **Psychiatric Comorbidities (N, %)** | | | | | | | |
| Anxiety Disorders | 197 | 15% | 93 | 15% | 104 | 15% | 0.846 |
| Mood disorders | 443 | 34% | 224 | 37% | 219 | 31% | 0.038 |
| Alcohol-related disorders | 126 | 10% | 48 | 8% | 78 | 11% | 0.045 |
| Substance-related disorders | 202 | 15% | 70 | 12% | 132 | 19% | <0.001 |
| Suicide and intentional selfinflicted injury | 83 | 6% | 37 | 6% | 46 | 7% | 0.707 |
| Screening/history of mental health and substance abuse | 253 | 19% | 113 | 19% | 140 | 20% | 0.499 |
| **Medical Comorbidities** | | | | | | | |
| Deyo Charlson Comorbidity Index (Mean, SD) | 0.9 | 1.7 | 0.7 | 1.3 | 1.0 | 1.9 | <0.001 |
| Number of unique primary 3-digit ICD-9 Diagnoses (Mean, SD) | 1.6 | 0.8 | 1.5 | 0.7 | 1.6 0 | .9 | 0.037 |

SD: standard deviation; ICD-9: International Classification of Disease, 9th Revision, Clinical Modification

**Table 2.** Hospitalization and Treatment Characteristics

| | All Patients N = 1312 | | Follow-Up Visit N = 611 | | No Follow-Up Visit N = 701 | | |
|---|---|---|---|---|---|---|---|
| | N/ Mean | %/SD | N/ Mean | %/SD | N/ Mean | %/SD | P-value |
| **Year of Admission (N, %)** | | | | | | | <0.001 |
| 2005 | 151 | 12% | 37 | 6% | 114 | 16% | |
| 2006 | 296 | 23% | 118 | 19% | 178 | 25% | |
| 2007 | 315 | 24% | 180 | 30% | 135 | 19% | |
| 2008 | 293 | 22% | 148 | 24% | 145 | 21% | |
| 2009 | 131 | 10% | 67 | 11% | 64 | 9% | |
| 2010/2011 | 126 | 10% | 61 | 10% | 65 | 9% | |
| **Length of Hospital Stay, days (mean, SD)** | 5.8 | 4 | 5.9 | 4.1 | 5.8 | 3.9 | 0.693 |
| **Admission Source (N, %)** | | | | | | | 0.036 |
| Emergency room | 595 | 45% | 296 | 48% | 299 | 43% | |
| Other/Unknown | 717 | 55% | 315 | 52% | 402 | 57% | |
| **Attending Physician Specialty (N, %)** | | | | | | | <0.001 |
| Psychiatry/Psychology | 510 | 39% | 274 | 45% | 236 | 34% | |
| Other/Unknown | 802 | 61% | 337 | 55% | 465 | 66% | |
| **Antipsychotic Treatment During Index Admission[a] (N, %)** | | | | | | | |
| Typical oral | 382 | 29% | 189 | 31% | 193 | 28% | 0.176 |
| Atypical oral | 1192 | 91% | 557 | 91% | 635 | 91% | 0.718 |
| Typical long-acting injectable | 225 | 17% | 114 | 19% | 111 | 16% | 0.176 |
| Atypical long-acting injectable | 96 | 7% | 50 | 8% | 46 | 7% | 0.261 |
| **Number of Days from last Inpatient Antipsychotic Administration to Discharge (mean, SD)** | 4.6 | 27.7 | 0.5 | 40.3 | 2.5 | 4.8 | 0.181 |
| **Pre-period Treatment and Healthcare Utilization** | | | | | | | |
| No antipsychotic treatment (N, %) | 242 | 18% | 66 | 11% | 176 | 25% | <0.001 |
| Treated with oral antipsychotics only[b] (N, %) | 841 | 64% | 419 | 69% | 422 | 60% | |
| Other antipsychotic treatment (N, %) | 229 | 18% | 126 | 21% | 103 | 15% | |
| Number of unique antipsychotics (all types) (mean, SD) | 2 | 1.8 | 2.4 | 1.9 | 1.7 | 1.6 | <0.001 |
| Psychiatric-related hospitalization (N, %) | 432 | 33% | 196 | 32% | 236 | 34% | 0.542 |
| Psychiatric-related ER Visit (N, %) | 495 | 38% | 202 | 33% | 293 | 0.001 | |
| Psychiatric-related outpatient office visit (N, %) | 10 | 1% | 5 | 1% | 5 | 1% | 0.827 |
| Community mental health organization visit (N, %) | 4 | 0% | 3 | 1% | 1 | 0% | 0.254 |

SD: standard deviation; [a]Adds to greater than 100% because some patients received multiple antipsychotic medication types during index admission; [b]Patient may have been treated with short-acting injectables.

**Table 3.** Hospital Characteristics

| | All Patients N=1312 | | Follow-Up Visit N=611 | | No Follow-up Visit N=701 | | |
|---|---|---|---|---|---|---|---|
| | N/Mean | %/SD | N/Mean | %/SD | N/Mean | %/SD | P-value |
| **Hospital Region (N, %)** | | | | | | | <0.001 |
| Northeast | 0 | 0% | 0 | 0% | 0 | 0% | |
| North Central | 257 | 20% | 168 | 28% | 89 | 13% | |
| South | 1048 | 80% | 442 | 72% | 606 | 86% | |
| West | 7 | 1% | 1 | 0% | 6 | 1% | |
| **Hospital Urbanicity (N, %)** | | | | | | | 0.445 |
| Urban | 1272 | 97% | 590 | 97% | 682 | 97% | |
| Rural | 40 | 3% | 21 | 3% | 19 | 3% | |
| **Teaching Status (N, %)** | | | | | | | 0.782 |
| Teaching | 16 | 1% | 8 | 1% | 8 | 1% | |
| Non-teaching | 1296 | 99% | 603 | 99% | 693 | 99% | |
| **Licensed Bed Size (N, %)** | | | | | | | <0.001 |
| 1-199 beds | 13 | 1% | 3 | 1% | 10 | 1% | |
| 200-299 beds | 481 | 37% | 270 | 44% | 211 | 30% | |
| 300-499 beds | 507 | 39% | 218 | 36% | 289 | 41% | |
| 500+ beds | 311 | 24% | 120 | 20% | 191 | 27% | |

SD: standard deviation

### Multivariable Results

The Cox proportional hazards modeling found no association between type of antipsychotic medication received during index admission and outpatient follow-up visit within 30 days post-discharge (Table 4). Several other factors were significantly associated with follow-up visit. Patients enrolled in a capitated insurance plan were significantly more likely to have a follow-up visit (AHR=1.31, 95% CI=1.05-1.63), as were patients who had an attending physician specializing in psychiatry/psychology during their index admission (AHR=1.30, 95% CI=1.08-1.56) and patients who had a greater number of unique antipsychotic medications (including LAI and oral) in the pre-period (AHR=1.16, 95% CI=1.11-1.21). Significantly less likely to have a follow-up visit were patients diagnosed with substance-related disorders (AHR=0.68, 95% CI=0.53-0.87) and those with increased scores on the Deyo Charlson Comorbidity Index, which indicates greater overall disease burden (AHR=0.92, 95% CI=0.87-0.98). The strongest predictor of a follow-up visit was year of index admission. Patients with index admissions in 2010/2011 were three times more likely to have a follow-up visit compared to patients with index admission in 2005 (AHR=3.03, 95% CI=1.99-4.61), controlling for other factors.

**Table 4.** Cox Proportional Hazards Model Results

| | Follow-up Visit | | |
|---|---|---|---|
| | **AHR** | **95% CI** | **P-value** |
| **Antipsychotic Medication Use During Index Hospitalization** | | | |
| Typical oral antipsychotic (vs. No) | 1.09 | 0.89-1.33 | 0.402 |
| Atypical oral antipsychotic (vs. No) | 0.98 | 0.72-1.33 | 0.884 |
| Typical long-acting injectable antipsychotic (vs. No) | 1.07 | 0.85-1.35 | 0.544 |
| Atypical long-acting injectable antipsychotic (vs. No) | 1.20 | 0.89-1.61 | 0.232 |
| Days from last in-hospital antipsychotic administration to index discharge | 0.997 | 0.995-0.999 | 0.002 |
| **Insurance Plan Capitated (vs. Not Capitated)** | 1.31 | 1.05-1.63 | 0.017 |
| **Attending Physician Specialty Psychiatry/Psychology (vs. Other)** | 1.30 | 1.08-1.56 | 0.005 |
| **Hospital Region (vs. Northeast/North Central)** | | | |
| South | 0.59 | 0.47-0.74 | <0.001 |
| West | 0.17 | 0.02-1.25 | 0.083 |
| **Pre-period Diagnosis of Substance-related Disorder (vs. No)** | .68 | 0.53-0.87 | 0.003 |
| **Pre-period Deyo Charlson Comorbidity Index** | 0.92 | 0.87-0.98 | 0.009 |
| **Number of Unique Antipsychotics Used During the Pre-period** | 1.16 | 1.11-1.21 | <0.001 |
| **Year of Index Admission (vs. 2005)** | | | |
| 2006 | 1.72 | 1.18-2.49 | 0.004 |
| 2007 | 2.95 | 2.07-4.21 | <0.001 |
| 2008 | 2.57 | 1.78-3.70 | <0.001 |
| 2009 | 2.59 | 1.71-3.93 | <0.001 |
| 2010/2011 | 3.03 | 1.99-4.61 | <0.001 |

AHR: adjusted hazard ratio; CI: confidence interval

## DISCUSSION

Of the 1,312 Medicaid beneficiaries with a schizophrenia-related hospitalization during which a LAI or oral antipsychotic medication was administered, only 47% had a follow-up psychiatric-related outpatient office or community mental health organization visit based on the HEDIS definition within 30 days of discharge. Compared to 2005, patients admitted in later study years were more likely to have a timely outpatient follow-up visit. Type of antipsychotic medication administered during hospitalization was not associated with follow-up visit. The data sources utilized provided the opportunity to evaluate inpatient drug use which is uncommon in claims-based analyses and allowed for the evaluation of trends in follow-up rates across multiple years. This combination of hospital data and claims data provided a thorough description of the patients and the factors which may affect timing of follow-up visit after discharge.

The proportion of patients receiving follow-up care is consistent with other analyses conducted using Medicaid claims.[5,6] Olfson, et al. evaluated 59,567 hospitalizations with schizophrenia as the first diagnosis in 2003 Medicaid claims.[5] The analysis found that within 30 days, 59% of hospitalizations were followed by an outpatient visit.[5] A similar analysis was conducted by Stein, et al. using 2004-2005 claims from a large Medicaid-managed behavioral health organization.[6] In Stein's study, 6,730 patients discharged from psychiatric hospitals were identified and the first hospitalization analyzed.[6] Stein reported that 49% of patients had follow-up care, defined as any specialty behavioral health service, within 30 days.[6]

Both the Olfson and Stein analyses were conducted over narrow time periods and therefore cannot provide sufficient information on trends in follow-up visits rate. In the current analysis, later index hospitalization discharge years were associated with a higher probability of follow-up visit. This may reflect an increased focus in recent years on improving the linkage between inpatient and outpatient care.[9] The importance of follow-up care has been highlighted by the inclusion of a measure for "follow-up after hospitalization for mental illness" in HEDIS.[7] Although their impact on behavioral healthcare quality to date has been mixed,[10] HEDIS measurements have positively impacted health practices in other disease areas.[11,12] Similar analyses with other data sources should be conducted to confirm the trend noted here.

Type of antipsychotic medication administered during inpatient hospitalization was not significantly associated with follow-up visit within 30 days of discharge. Patients administered an atypical LAI during hospitalization tended to be more likely to have a follow-up visit than those who were administered other types of antipsychotics, but this finding was not statistically significant. In Olfson's analysis, the type of antipsychotic medications received in the 90 days prior to hospitalization was a strong predictor of followup care.[5] Compared to patients who used no antipsychotic medications, patients with evidence of depot medication were nearly three times as likely to have a follow-up visit within 30 days of discharge and patients with evidence of oral medication use were nearly twice as likely to have a follow-up visit.[5] Olfson and colleagues hypothesized that depot medication use may indicate strong connections to outpatient care and therefore greater probability of follow-up after hospitalization.[5] It is possible that use of LAIs in the inpatient setting does not reflect stability of a patient's outpatient care, but rather the prescribing preferences of physicians or hospital standards, and therefore the association between inpatient LAI administration and outpatient follow-up visit is weakened. Inpatient receipt of atypical LAIs has been associated with other health outcomes. Lafeuille and colleagues found that among patients initially treated with oral antipsychotics in the inpatient setting, those who were switched to atypical LAIs during a subsequent hospitalization had lower rehospitalization and ER visit rates compared to patients who received oral antipsychotics only.[13]

The results of this study suggest a benefit for hospitalized schizophrenia patients who receive care from a psychiatrist/psychologist during their hospitalization, as compared to other specialists or general practitioners. It is possible mental health specialists are better equipped or trained to coordinate care transitions. A similar result was reported by Olfson, who found that patients treated at psychiatric hospitals versus general hospitals, and those living in areas with a higher numbers of psychiatrists, were more likely to have a followup visit within 30 days after discharge.[5] Also more likely to have a follow-up visit were patients enrolled in capitated insurance plans. Capitated Medicaid plans differ widely,[14] so the explanation for this is not immediately apparent and further research is needed to explore the reason behind this finding. It could be related to financial incentives on capitated plans to decrease length of stay and reduce readmissions, which may increase plans' focus on providing intensive discharge planning services or incentive plans to remove barriers to outpatient services, such as long wait times for follow-up visits.[15] Olfson reported contradictory results, finding that patients in fee-for-services plans had a greater probability of follow-up visit.[5] More research is needed regarding this variable though varying data years and Medicaid states included in the data may account for this discrepancy.

Finally, patients with schizophrenia and comorbid substance abuse disorder were less likely to have a followup visit within 30 days, suggesting a population that may need additional attention among both discharge planners and outpatient care providers. Other analyses have found similar relationships between substance abuse and follow-up care.[5,6] These patients represent a marginalized and stigmatized population often out of reach of healthcare providers. An inpatient admission represents an important contact with healthcare which should serve as an opportunity to initiate regular care.

This analysis has limitations. As with all observational analyses, certain variables remained unmeasured, such as details of discharge planning, clinical measures of disease severity, patients' living arrangement and caregiver information, which may be associated with follow-up visit. The potential for misclassification of type of antipsychotic medication use during hospitalization, covariates, or outcomes is present as these were identified through administrative data which are subject to data coding limitations, such as reliance on route of administration variables and data entry error. Misclassification of the study outcome may have occurred if patients had a follow-up visit that was not billed to Medicaid because these visits would not appear in the Medicaid claims database. This analysis was limited primarily to psychiatric care provided in general hospital psychiatric units, as psychiatric inpatient treatment provided in psychiatric hospitals with more than 16 beds is not reimbursed by Medicaid. Therefore, while the results may differ for patients receiving care in psychiatric hospitals, the care received by this patient population in general hospitals may be typical of what Medicaid-insured individuals receive. The patient sample was a convenience sample that may not be representative of the overall Medicaid population or generalizable to commercially insured, Medicare, or uninsured populations.

Potential future analyses include an analysis of first hospitalizations for patients with schizophrenia when more real-world data is available for atypical LAI, as comparing only patients experiencing first hospitalization may reduce residual confounding by clinical factors. Additionally, a similar analysis could be conducted to determine factors associated with readmission within 30 days.

## CONCLUSION

Less than half of patients had a follow-up visit within 30 days of discharge in this analysis of Medicaid enrollees hospitalized and treated for schizophrenia, though the rates of follow-up visit have increased since 2005. This finding highlights the need for continued efforts to promote effective linkage to outpatient care for patients hospitalized and treated for schizophrenia. Patients with schizophrenia suffering from comorbid substance abuse disorders represent a high-risk population requiring particular attention.

## DECLARATION OF COMPETING INTERESTS

This study was funded by Janssen Scientific Affairs. JP is an employee of Janssen Scientific Affairs. AF, DS, and ZC were employees of Truven Health Analytics at the time this analysis was conducted, which received funding from the sponsor to conduct this study.
